# Supplementary figures and images for: Syndecan-1 Plays a Role in the Pathogenesis of Sjögren’s Disease by Inducing B-Cell Chemotaxis through CXCL13–Heparan Sulfate Interaction
Source: Int J Mol Sci. 2024 Aug 29;25(17):9375. doi: 10.3390/ijms25179375 (PMC11394922; doi:10.3390/ijms25179375)

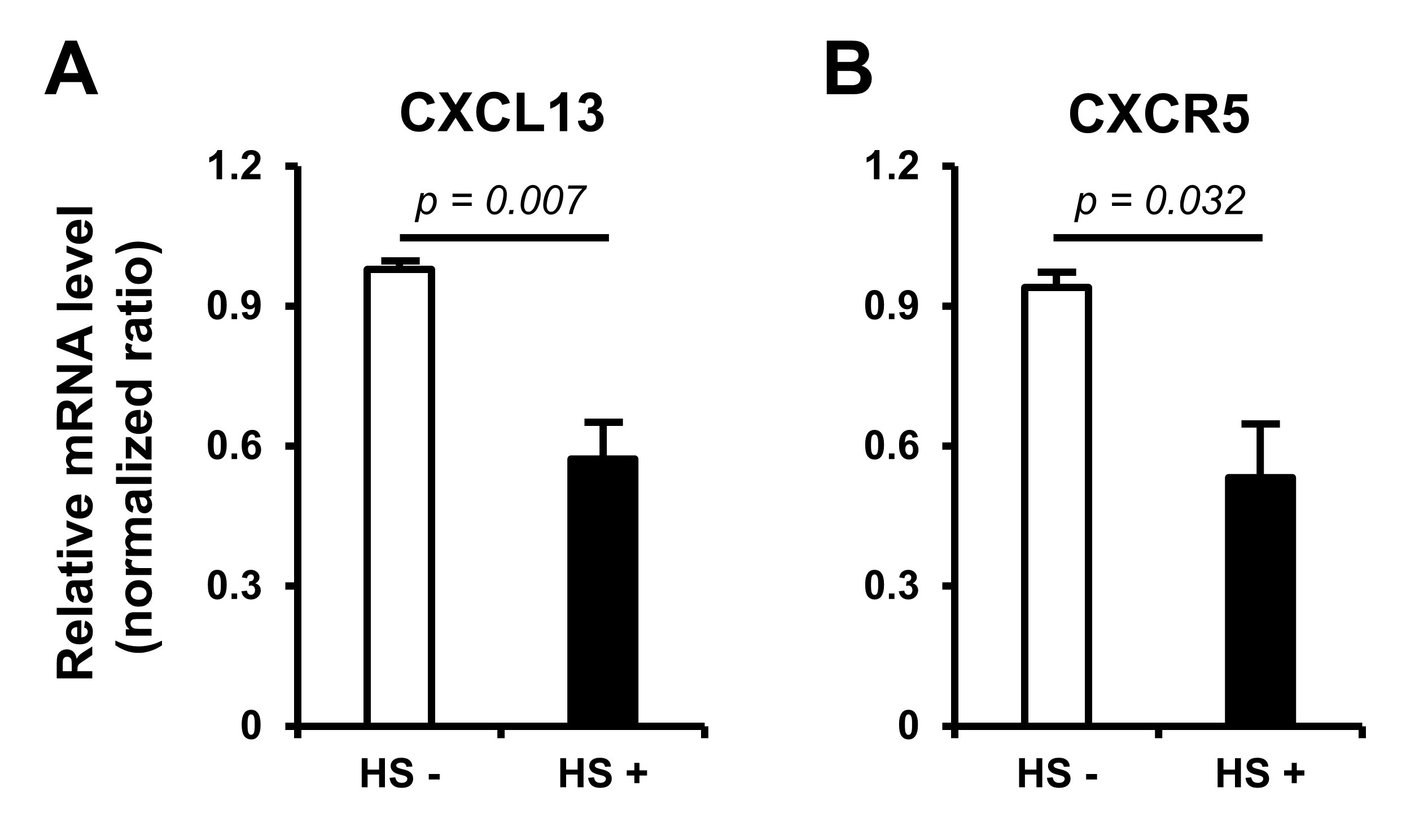

Supplement: Supplementary file 1 [file ijms-25-09375-s001.zip › Figure S1. RT-PCR for CXCL13 and CXCR5-Revised.jpg]

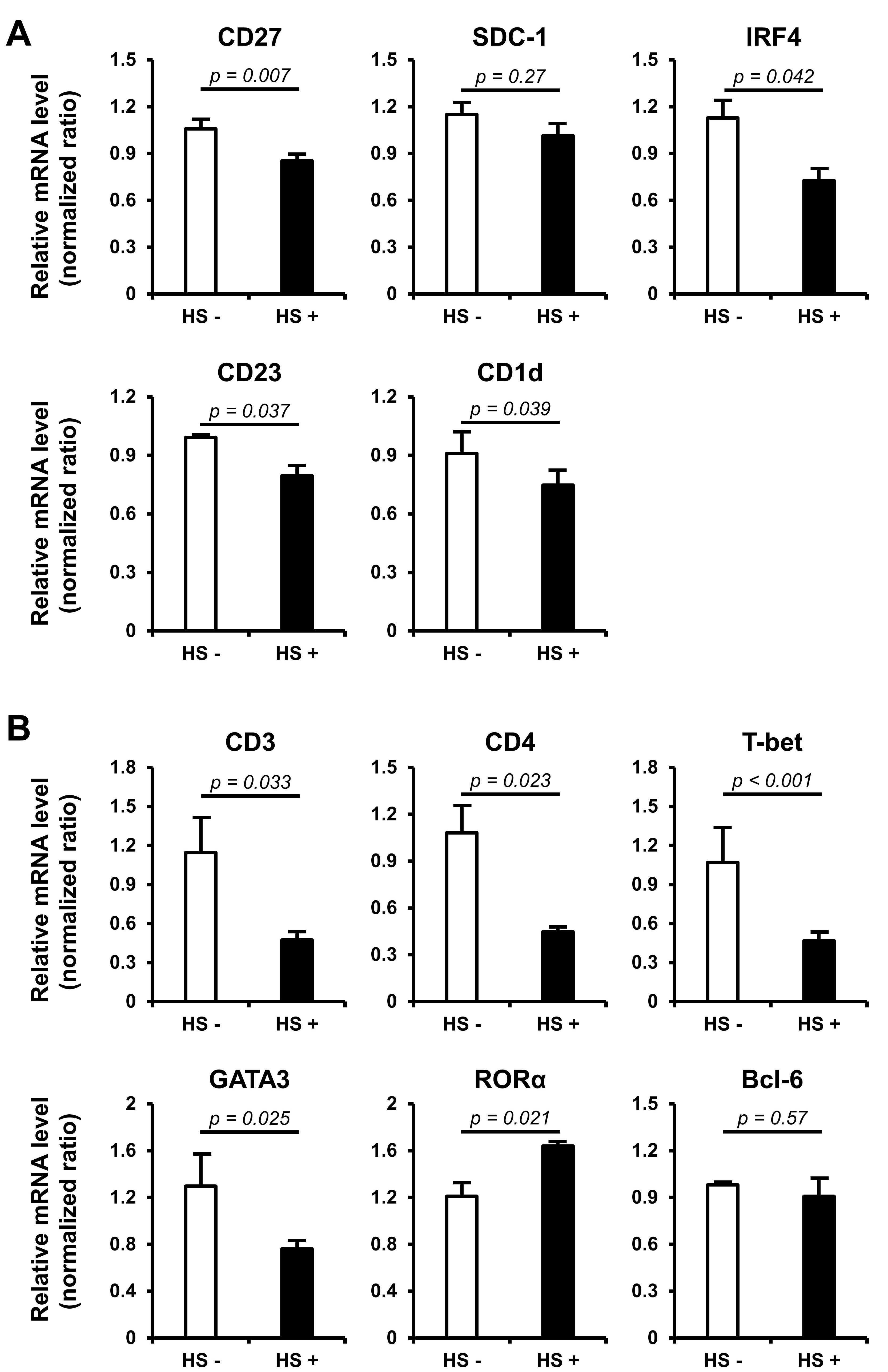

Supplement: Supplementary file 1 [file ijms-25-09375-s001.zip › Figure S2. RT-PCR for subtypes of B and T cells.-Revised.jpg]
